# Revisiting the need for radiation output measurements after X‐ray tube replacement in computed tomography

**DOI:** 10.1002/acm2.13359

**Published:** 2021-07-19

**Authors:** Paul J. Stauduhar, A. Kyle Jones

**Affiliations:** ^1^ Department of Imaging Physics The University of Texas MD Anderson Cancer Center Houston TX USA; ^2^ Present address: Care Continuity Dallas TX 75247 USA

**Keywords:** computed tomography (CT), image noise, output, quality control (QC), statistical process control

## Abstract

**Purpose:**

State regulations require that CT radiation output be measured using a dosimeter after major service. The most common major service is tube replacement. We hypothesized that historical QC data could be used instead to determine if output measurements are necessary, potentially reducing the need for output measurements that require an on‐site visit by a qualified medical physicist.

**Methods:**

Records of 65 original equipment manufacturer (OEM) tube replacements were reviewed to determine with what frequency output was outside the manufacturer's specifications. The previous 7 days of historical quality control (QC) data prior to a tube change was used to establish a baseline mean noise level and 95% inferential confidence intervals (ICIs) about the mean. This was compared to an ICI constructed using 7 days of QC data post‐tube change and the region of indifference. Different methods for acquiring samples of image noise were compared using a single factor analysis of variance (ANOVA).

**Results:**

None of the 65 tube replacements reviewed in this study resulted in an output change that exceeded the manufacturer's specifications. In all but one case, the results of the ICI analysis matched the measured output results. In the single case where results were discordant, the mean image noise was slightly higher after the tube change, which may have indicated the need for a larger sample size or service unrelated to the X‐ray tube, for example, system calibration. The method used to sample image noise did not significantly affect the calculated mean noise.

**Conclusions:**

This review of historical OEM tube replacement data indicated the likelihood of output falling outside manufacturer specifications is low. Considering this, it is likely that using QC data from programs required by regulation and the American College of Radiology (ACR), medical physicists can reliably verify radiation output stability remotely instead of making measurements using a dosimeter.

## 1 INTRODUCTION

State regulations^1^, ^2^, ^3^, ^4^, ^5^, ^6^ (this is not an exhaustive list) require measurement of radiation output using a calibrated dosimeter for computed tomography (CT) scanners after major service, including replacement of the X‐ray tube. We propose that radiation output measurements using a dosimeter after major service are unnecessary in most cases. All major manufacturers of CT equipment have requirements for daily quality control (QC) in their instructions for use, state regulations require such QC to be performed,^3^, ^4^, ^6^ and federal regulation requires that the manufacturers provide a phantom to be used for such QC.^7^ The American College of Radiology (ACR) also requires accredited facilities to perform, monitor, and review daily QC.^8^ These QC data are used to benchmark the baseline system performance and identify concerning trends or abnormalities in system performance. We propose that it can also be used to assess radiation output after X‐ray tube replacement without performing radiation output measurements with a dosimeter.

## 2 MATERIALS AND METHODS

For filtered backprojection image reconstruction, image noise is predictably related to radiation output:

(1) $$Noise\propto \frac{1}{\sqrt{\mathrm{Output}}}.$$

Therefore, when using the same tube potential (kV), reconstruction kernel, image thickness, and bowtie filter, image noise is inversely proportional to the square root of the radiation output. If the same mAs is used to acquire QC images, then the image noise is proportional to radiation output in mGy/mAs, which is exactly what is measured with a dosimeter after major service.

**FIGURE 1:**
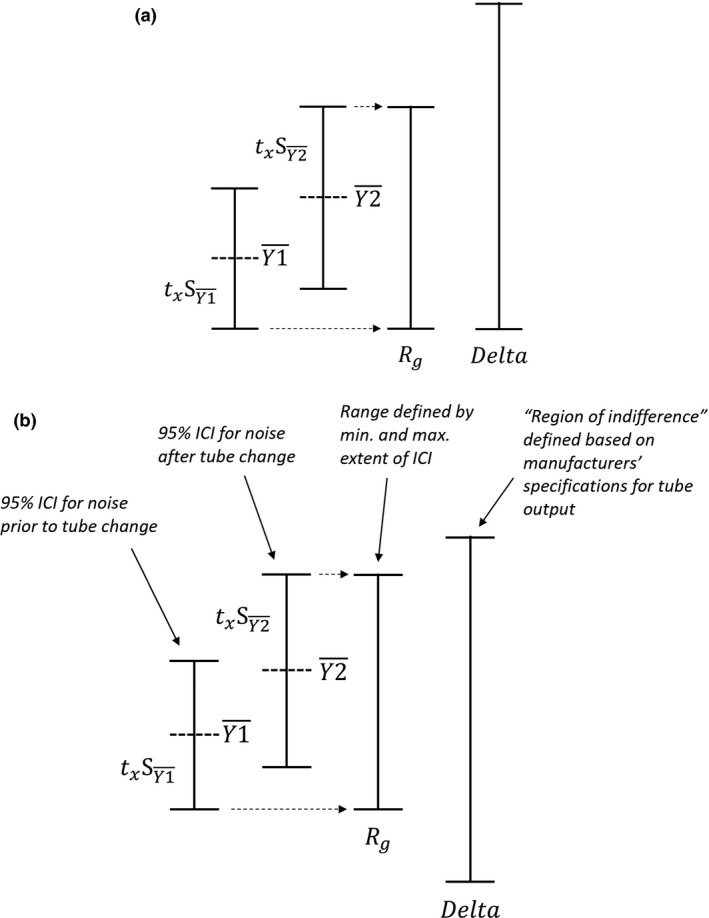
(a) Conceptual illustration of the use of inferential confidence intervals (ICIs). *R_g_* represents the range of the confidence intervals for the two samples being compared. *Delta* represents the “range of indifference,” or the range of values that are acceptable based on theoretical considerations, in this case the tolerance on radiation output as specified by the manufacturer. (b) Adaptation of this method to the problem of assessing radiation output after X‐ray tube change by “centering” *Delta* about the pre‐tube change noise mean. Note the “centering” is asymmetric because of the relationship between image noise and radiation output, as discussed in the text

The concept of statistical equivalence was applied using inferential confidence intervals (ICIs),^9^ based on the idea that if the *range* (*R_g_*) of the 95% confidence intervals (CIs) about the means of two samples is entirely contained within some range of no consequence (*Delta*),^10^ determined based on “substantive theoretical considerations,” then the samples are statistically equivalent (Figure 1a). In the context of CT X‐ray tube replacement, *Delta* was defined using the manufacturer's specified tolerances for X‐ray output. We used the method of Tryon,^9^ which was adapted from the method of Dunnett and Gent^10^ to use *R_g_* instead of calculating the 95% CI about the difference of the means. The desired (e.g., 95%) ICI about the mean of each sample Y, in this case the image noise pre‐tube change and post‐tube change, is given by the sample mean plus or minus *t* standard errors of the mean:

(2) $$\bar{Y}\pm t_{\alpha /2}S_{\bar{Y}}=\bar{Y}\pm t_{\alpha /2}\frac{S}{\sqrt{N}},$$

where $\bar{Y}$ is the sample mean; $t_{\alpha /2}$ is the critical t value selected based on the sample size *N*, degrees of freedom, and desired confidence level; $S_{\bar{Y}}$ is the standard error of the mean; and *S* is the sample standard deviation. Using this method, when comparing two means the standard error of the difference of the means is expressed in terms of the standard error of each of each group, a factor labeled *E* by Tryon^9^:

(3) $$E=\frac{\sqrt{S_{{\bar{Y}}_{1}}^{2}+S_{{\bar{Y}}_{2}}^{2}}}{S_{{\bar{Y}}_{1}}+S_{{\bar{Y}}_{2}}},$$

where a subscript 1 indicates the standard error of the mean for pre‐tube change image noise and a subscript 2 indicates the standard error of the mean for post‐tube change image noise. Thus the final expression for the CI is:

(4) $$\bar{Y}\pm Et_{\alpha /2}\frac{S}{\sqrt{N}}.$$

Nonoverlap of two ICI calculated using Equation (4) is equivalent to statistical difference determined using a standard *t*‐test.^9^ For further description of the derivation of ICI, the reader is referred to Ref. 9. Tryon's method was adapted to the problem of radiation output assessment after X‐ray tube change by setting *Delta* equal to the manufacturers' specified limits for X‐ray output and “centering”^*^ *Delta* about the mean of the pre‐tube change image noise (Figure 1b). This was necessary because the benchmark in this case was the baseline image noise prior to the X‐ray tube change, while Tryon's method aligns the lower bound of *Delta* with the lower limit of the lesser mean. Other criteria for setting *Delta* can be used, including internal QC limits.

The records of 65 original equipment manufacturer (OEM) X‐ray tube replacements spanning an 8‐year period at a single institution were reviewed retrospectively to determine with what frequency radiation output measured with a dosimeter failed to meet the manufacturers' specifications. The dataset included a total of nine scanner models from two manufacturers (General Electric, Waukesha, WI: HD750, VCT, LS16, LS8, LS+, LS QX/i; Siemens Healthineers, Malvern, PA: Sensation Open, Sensation 16, Sensation 64). Radiation output measurements (central CTDI_100_) performed with a calibrated dosimeter within 1 week of X‐ray tube replacement were compared to the most recent prior radiation output measurement, typically the measurement from the previous annual performance evaluation. A minimum of 7 days of historical daily QC data pre‐ and post‐tube change was available from 63 of the 65 affected scanners, and these data were analyzed using the proposed ICI method, with *Delta* calculated based on the pre‐tube change mean image noise using a ±15% radiation output tolerance^†^, the most strict of any of the specifications for the scanners included in the study. These daily QC data were acquired using identical protocols (for a given manufacturer) across the entire install base evaluated in this study.

As several states required that radiation output measurements to be performed within 30 days of an X‐ray tube change,^1^, ^3^, ^6^ different methods for acquiring 30 samples of image noise after an X‐ray tube change were evaluated, including one measurement of the noise in a single image from 30 days of QC data, a single image from 30 scans of the phantom on a single day, and 30 measurements made in different images of 3 scans of the phantom on a single day. The methods were compared using a single factor analysis of variance (ANOVA).

## 3 RESULTS

**FIGURE 2:**
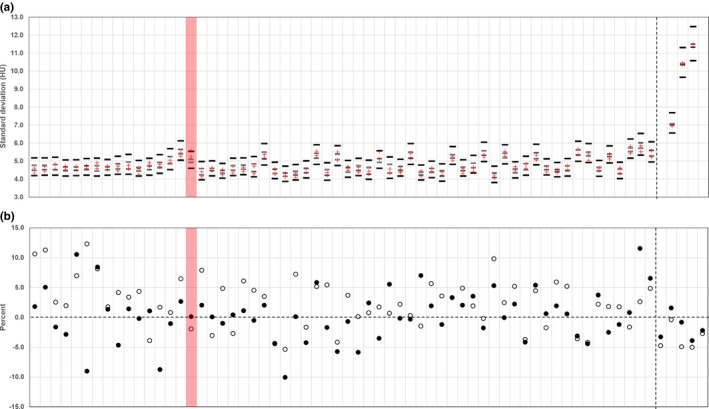
Results of the retrospective analysis of 65 X‐ray tube replacements. The five Siemens CT scanners, which used different techniques for daily QC, are grouped on the right side of the plot, marked by the dashed vertical line. There was no historical image noise data available for two X‐ray tube replacements on the Siemens CT scanners. Otherwise, one sample of the noise was measured in 7 days of historical QC data both before and after the X‐ray tube change. The red shading identifies the one X‐ray tube replacement for which the range of the pre‐ and post‐tube change confidence intervals was not contained within the region of indifference, calculated based on the manufacturers' specified expected variation in X‐ray output. (a) Image noise measurements, with solid horizontal bars representing the limits of *Delta*, determined based on the manufacturers' specified limits for changes in X‐ray tube output. Open horizontal bars represent the 95% inferential confidence interval (ICI) for image noise prior to a tube change, “+” represents the 95% ICI for image noise after a tube change. (b) Percent change in radiation output relative to the most recent previous output measurement (typically the most recent annual performance evaluation) using a calibrated dosimeter(●); percent error in radiation output compared to the manufacturers' specified radiation output (○)

For all 65 X‐ray tube replacements evaluated, measured radiation output after tube replacement was not only within the manufacturers' specified limits, but was within the manufacturers' specified expected variation (Figure 2). In all but one case, this result was confirmed by statistical analysis using ICI. For the one exception, a GE VCT, measured image noise was 8.0% higher after the X‐ray tube change, corresponding to a theoretical change in X‐ray output of approximately −17%, still well within the maximum limits of +/‐ 40% allowed by the manufacturer. However, the radiation output measured with a calibrated dosimeter post‐tube change was only 0.09% higher than the pre‐tube change baseline, and differed by −2.0% from the manufacturer‐specified radiation output. When 14 days of QC data before and after the X‐ray tube change were evaluated for this exception, the post‐tube change ICI was contained within *Delta* but still did not overlap the pre‐tube change ICI. This may indicate that the measured increase in image noise after the X‐ray tube change was unrelated to radiation output, for example, indicating the need for CT system calibration.

Considering all 65 tube replacements evaluated, mean radiation output post‐tube change was almost identical to mean radiation output at baseline (Table 1). Mean image noise was more than twice as likely to be lower after a tube change rather than higher, however, the mean difference was very small, only −0.6%. The mean and variance of 30 image noise samples were not significantly different among the three methods used to obtain 30 samples (*F* = 1.71, *p* = 0.18).

**TABLE 1:** Summary of review of historical X‐ray tube replacements

Mean percent change in radiation output be measured using a dosimeter compared to pre‐tube change baseline (mean magnitude of the percent change)	Percentage of tube changes after which radiation output increased	Mean percent error in radiation output measured using a dosimeter compared to the manufacturers' specifications (mean magnitude of the percent change)	Percentage of tube changes after which radiation output was higher than the manufacturers' specifications	Mean percent change in image noise compared to pre‐tube change baseline (mean magnitude of the percent change)	Percentage of tube changes after which image noise decreased*
0.20% (3.2%)	52% (34/65)	2.0% (4.0%)	69% (45/65)	−0.59% (1.8%)	65% (41/63)

* There were no historical image noise data available for two X‐ray tube replacements, hence the denominator of 63.

## 4 DISCUSSION

The results of this retrospective analysis of X‐ray tube changes indicated that, for OEM X‐ray tubes and service, changes in X‐ray tube radiation output that exceeded the manufacturers' specified expected variation were rare and radiation output and image noise were within expected limits after tube replacement. Considering the rarity of events that would be expected to result in actionable changes in radiation output, and the relative frequency of X‐ray tube changes (during the last year of retrospective data collection, 25% [10/40] of the CT scanner base evaluated in this study required an X‐ray tube change), it is reasonable to propose an alternative to the current state of practice of making measurements of radiation output using a dosimeter. Even when using only 7 days of daily QC measurements of image noise before and after an X‐ray tube change, assessment using ICI matched the radiation output measurements with one exception when compared to expected variation (but in all cases when compared to maximum variation). The single exception might have indicated a need for further follow‐up for an issue unrelated to X‐ray tube output, as increasing the sample size in both the pre‐ and post‐tube change period produced a similar result.

As the method used to sample image noise did not significantly affect the calculated image noise distribution, a protocol can be developed to allow for assessment of radiation output within the 30‐day window required by many states. While *E* is minimized when the standard errors of the two means are equal, the size of the 95% ICI depends more strongly on the standard error of the mean, which can be reduced using more samples, that is, more historical QC data from the pre‐tube change period. While not exactly applicable to this method, but still instructive, to detect a 15% change in X‐ray tube output using image noise measurements with 80% power at a Type I error rate of 0.05, 12 samples in each of the pre‐ and post‐tube change period would be required. Alternatively, using 30 samples in each of the pre‐ and post‐tube change period would detect the change with 99% power.

If the proposed method is to be used, it is imperative that standard QC protocols and methods be used, including positioning of the phantom within the field of view and location of ROI placement. Automated QC analysis methods can greatly simplify this process. Crossover analysis should be performed after software updates that affect reconstruction kernels.

One other caveat should be mentioned––when using the proposed method, it is possible that the 95% ICI for the mean image noise pre‐ and post‐tube change may not overlap yet *R_g_* still be contained within *Delta*. This is because the tolerance used for change in radiation output was based on a percent change from baseline, and not a significant difference between pre‐ and post‐tube change data. If this occurred, the upper or lower limit of the post‐tube change ICI would be very close to the limits of *Delta*. Considering that the proposed method is intended to be used to determine if radiation output measurements using a dosimeter are necessary and should therefore err on the side of being conservative, we propose that if the 95% ICI for pre‐ and post‐tube change image noise do not overlap then radiation output measurements using a dosimeter should be performed.

This study did have limitations. None of the X‐ray tube replacements studied involved a non‐OEM X‐ray tube, which may affect the rate of X‐ray tube changes resulting in radiation output changes that exceed specifications. However, the proposed method can be used to identify these changes, just as it can for OEM X‐ray tubes. Other considerations include verification of scanner‐reported dose indices and collimated beam width. Verification of scanner‐reported dose indices, for example, CTDI_vol_, may also be part of routine follow‐up of X‐ray tube changes. The reported CTDI_vol_ may not match the measured CTDI_vol_ if X‐ray tube radiation output has changed, or if lookup tables are not updated after an X‐ray tube change, if applicable. However, it is simple to have the technologist performing daily QC record the reported CTDI_vol_ for comparison. Finally, the proposed technique is likely insensitive to changes in collimated beam width, however, these are often detected automatically by the scanner (for scanners that use mechanically variable apertures) or their impact is manifested as artifacts in QC images. Furthermore, some manufacturers used fixed X‐ray aperture plates, and no changes in collimated beam width would be expected after an X‐ray tube change. Alternatively, the technologist can easily be trained to acquire data to measure the collimated beam width using radiochromic film, and these data can be sent to the qualified medical physicist for remote review.

## 5 CONCLUSION

Image noise in daily QC data can be used to determine if radiation output measurements using a dosimeter are necessary after an X‐ray tube change. An Excel (Microsoft, Redmond, WA) template that implements the approach used in this study has been provided in the supplemental materials accompanying this article online.

## 6 AUTHOR CONTRIBUTION STATEMENT

Both the authors met the following criteria: substantially contributed to the conception or design of the work, acquisition, analysis, or interpretation of the data; drafted the work or revised it critically for important intellectual content; approved the final version to be published manuscript; and agreed to be accountable for all aspects of the work in ensuring that questions related to the accuracy or integrity of any part of the work are appropriately investigated and resolved.

## CONFLICT OF INTEREST

Neither of the authors has anything to disclose.

## Supporting information

Supplementary Material

## Data Availability

Data available on request from the authors.

## ENDNOTES

^*^
Centering in this case was asymmetric, as taking the reciprocal of the square root of the maximum allowed change in radiation output (15%, in this case) produced limits of −6.7% to 8.5% for image noise.

^†^
The Technical Reference Manual (TRM) for GE specifies an expected variation of ±15% and a maximum variation of ±40%; the System Owner Manual for Siemens specifies a maximum variation of ±30%, while the tolerance for the CTDI Constancy test is set at ±20%.
